# Do strigolactones play a role in the ascent and attachment behavior of *Pisum sativum*?

**DOI:** 10.1080/15592324.2024.2447455

**Published:** 2024-12-30

**Authors:** Bianca Bonato, Tom Bennett, Silvia Guerra, Sara Avesani, Umberto Castiello

**Affiliations:** a Department of General Psychology, University of Padova, Padova, Italy; b Faculty of Biological Science, University of Leeds, Leeds, UK

**Keywords:** Strigolactones, plant behavior, kinematics, circumnutations, pea plant, climbing plant, *Pisum sativum*

## Abstract

Strigolactones (SLs) are signaling compounds made by plants. They play a crucial role in acting as long-distance signals from root to shoot to coordinate shoot growth with root environmental conditions. Here, we test whether and how SLs play a role in the climbing behavior of pea plants by studying the circumnutation of the tendrils using three-dimensional (3D) kinematical analysis. To assess this, we compare the typical behavior of *P. sativum*, a wild-type plant that produces and perceives SLs, with mutants defective in SLs synthesis or signaling, known as *ramosus*(*rms*) mutants. The results indicate that mutant plants seem unable to locate and grasp a potential support. Their movement appears to be disoriented and much less energized. We contend that this research opens new avenues for exploring SLs’ role in plant behavior, a novel lens through which the role of SLs in root-to-shoot communication can be observed and analyzed.

## Introduction

Plants produce and release a variety of chemicals into the environment, including primary and secondary metabolites.^1^ Among secondary metabolites, strigolactones (hereafter referred to as SLs) are notable examples of rhizosphere signaling molecules and represent a novel class of plant hormones with an array of biological functions being continuously uncovered.^2^

The initial understanding of SLs was focused on their role as chemical signals that facilitate root colonization by recruiting arbuscular mycorrhizal fungi, which form symbiotic relationships with plants. Recently, SLs have been recognized as endogenous plant hormones that regulate various aspects of plant growth and development, such as root architecture, secondary stem, and branch growth, and senescence.^3,4,5^ Moreover, several studies suggest that SLs play a critical role in plant responses to biotic and abiotic stresses, transmitting signals from the roots to the shoots.^6–10^

This growing body of research has spurred a surge of interest in SLs and catalyzed a wide range of studies across multiple disciplines and plant species on the various biological functions and implications of these hormones.^1,11^ For instance, in *Pisum sativum* (hereafter referred to as *P. sativum*), SLs not only play a role in plant growth and development,^1,11^ but also function as plant–plant signaling molecules, facilitating plant communication and neighbor detection.^12^ Further, it should be remembered that SLs allow communication with other organisms indicating ecological significance.^13^ SLs could play a role as host recognition signaling molecules for arbuscular mycorrhizal fungi^14–16^ and as rhizosphere signals for seed germination in parasitic weeds.^13,17,18^

*P. sativum* is an annual climbing plant (not self-supporting) that locates structural elements in its surroundings to grow vertically and access light, which is fundamental for its survival. Its ascent is characterized by circumnutation, a rotatory growth movement pattern first described by Darwin & Darwin in a large variety of plant species.^19^ Circumnutation is a helical organ movement that varies based on the magnitude of trajectory (amplitude) outlined by the organ tip; duration of one cycle (period); circular, elliptical, pendulum-like, or irregular shape; and clock- and counterclockwise direction of rotation.

Recently, circumnutation has been well characterized in pea plants via 3D kinematical analysis (e.g.^20^ This body of research suggests that the patterning of circumnutation is highly flexible, goal-directed, and sensitive to the structural features of potential supports^21,22^ as well as different contexts.^23,24^ For these modulations to occur, it appears critical that the support is detected underground. The plants’ tendrils appropriately scale kinematics only when the support is available to the root system but not when it is only available above-ground.^25^ A communication pathway between the roots and the aerial parts of the plant is thus necessary for the proper development of the branches and the stem,^25,26^ implying root-shoot signaling. Overall, these results suggest that the coding of a support’s features is achieved via a functional equilibrium subtended by crosstalk between the underground and the aerial components of the plant.^26–29^

Of interest here is that SLs play a crucial role in regulating plant growth and development at the shoot level, acting as long-distance signals from root to shoot to coordinate shoot growth with root environmental conditions.^30^ SLs are known primarily for their inhibitory effect on bud growth,^31,32^ yet they also promote the growth of specific tissues, such as the interfascicular cambium, contributing to vascular and tissue development.^33^ Beyond their role in bud regulation, SLs are integral to root development and demonstrate complex interactions with other hormonal pathways. SLs also play roles in regulating root growth, although these are rather variable between species.^34,35^ SLs exuded into the rhizosphere have also been found to act as intra-plant signals, that allow plants to modulate their growth to the presence of neighboring plants.^12^ In summary, SLs emerge as pivotal regulators of plant morphology, impacting both above- and below-ground structures through complex crosstalk with auxin and other hormones.

Here, we capitalize on this knowledge to test whether and how SLs play a role in the climbing behavior of pea plants. To assess this, we compare the typical behavior of *P. sativum*, a wild-type plant that produces and perceives SLs, with mutants defective in SL synthesis or signaling, known as *ramosus* (*rms*) mutants. Specifically, we examine *rms1–1* mutants lacking SL synthesis due to a mutation of the CCD8 enzyme^36^ and *rms3–1* mutants, which are unable to perceive SL due to a mutation of α/β hydrolase enzymes (see.^12^). Their movement was video recorded and stored for subsequent 3D kinematic analysis.

Overall, we hypothesize differences in movement patterning when comparing SL gene-mutant plants with wild-type plants. This could be due to the inability of SL mutants to coordinate their shoot behavior in response to the detection of a potential support through the root system^25,26^ because of the lack of SL to act as root-to-shoot signals.^30^ In this case, SL biosynthesis and signaling mutants would be expected to behave similarly. Alternatively, since SL act as rhizosphere signals that allow plants to sense aspects of the physical and biological environment,^12^ a lack of SL might alter the ability of plants to detect the presence of a potential support through the root system.

## Material and methods

### Subjects

**Figure 1:**
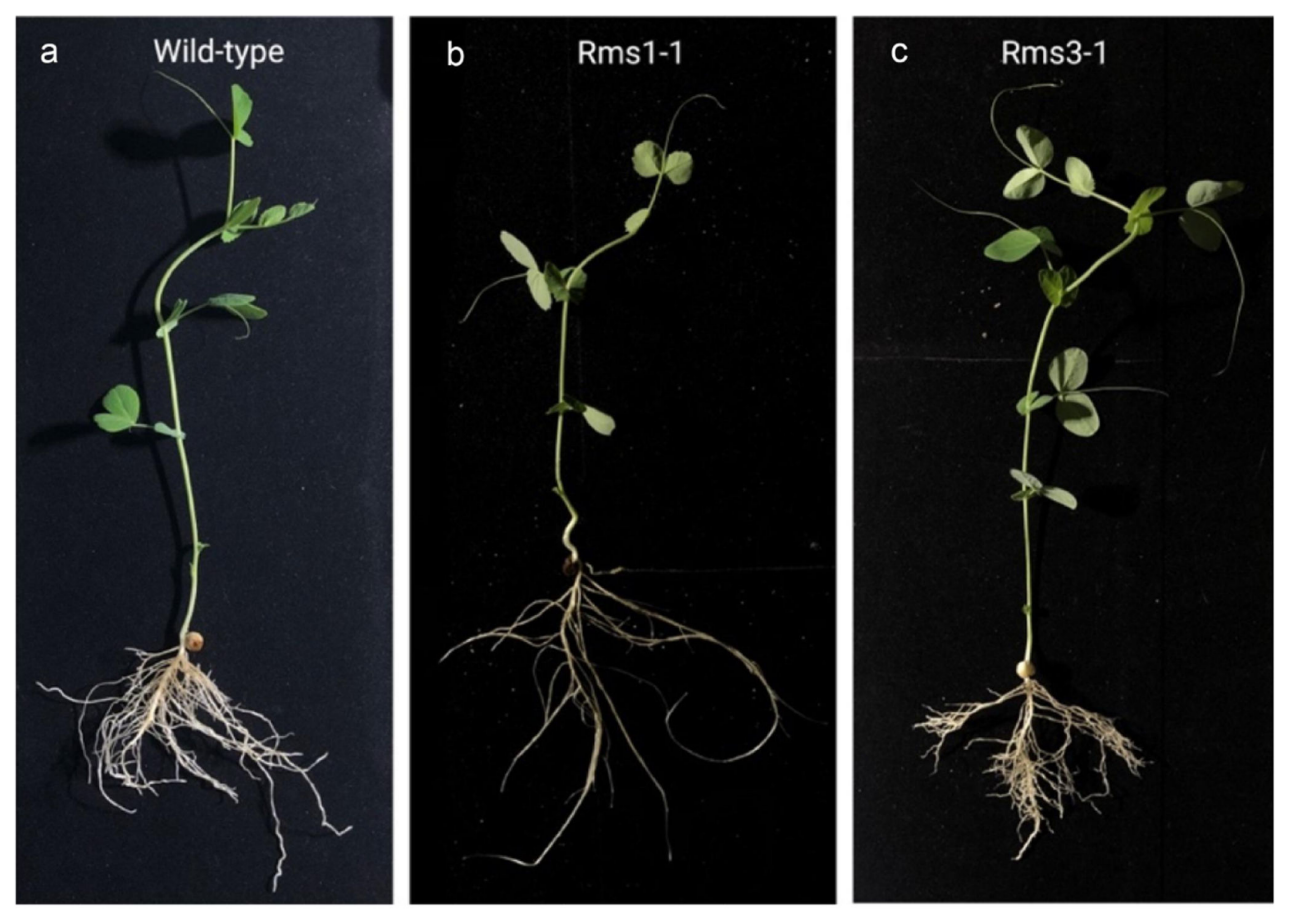
Photographs of representative plants for the three genotypes of *P. sativum* selected for the present study. Panel a: a representative plant for the wild-type genotype, *P. sativum* L. Panel b: a representative plant for the *rms1–1* mutant plant (L77 background). Panel c: a representative plant for the *rms3–1* mutant plant (Torsdag background) condition.

For this study, we selected eight (i) *P. sativum* L. wild types; (ii) eight *P. sativum* L. *rms3–1* mutant plants (Torsdag background); and (iii) eight *P. sativum* L. gene mutant plants, *rms1–1* (L77 background),^37,38^ as represented in Figure 1.

### Type of support

The support was a wooden pole 60 cm in height (6 cm underground and 54 cm above ground) and 0.5 cm in diameter.

### Germination and growth conditions

**Figure 2:**
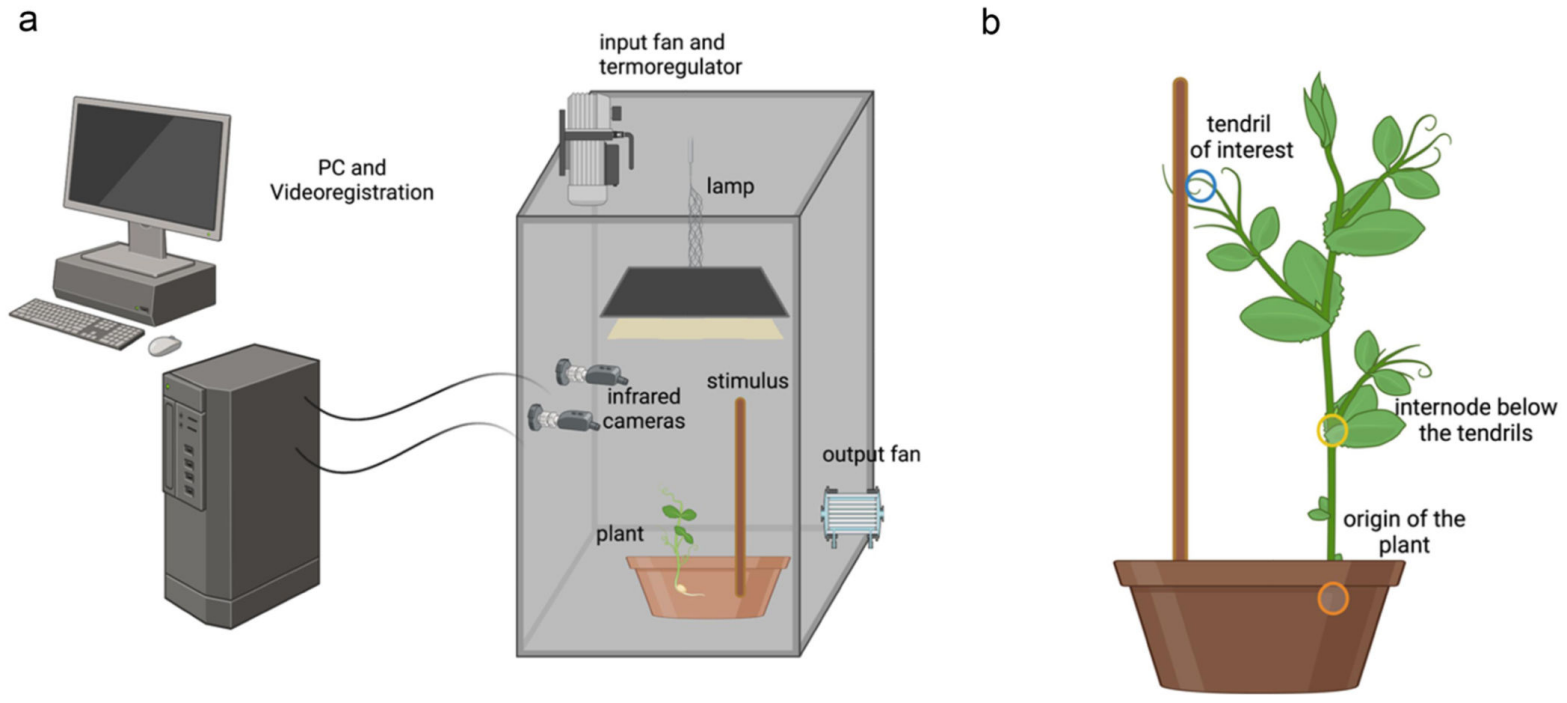
Graphical representation of the experimental setup (panel a) and the plant anatomical landmarks of interest (panel b). In panel b, the orange circle represents the origin of the plant (the point at which the plant emerges from the soil), the yellow circle represents the internode of the plant (the node below the tendril of interest), and the blue circle represents the tendril. The yellow and orange circles serve as a reference for the stems of the plants.

The seeds were germinated for five days on a filter paper strip soaked with water at 1.5 cm from each other and 0.5 cm from the top of the strip. Seed orientation showed the hilum and micropyle oriented downward. Then, the healthy and same-rate-height sprouts were selected and planted in a plastic pot of 20 cm in diameter and 20 cm in height. The pots were filled with silica sand (type 16SS, dimension 0.8/1.2 mm, weight 1.4). At the beginning of each treatment, the pots were watered and fertilized using a half-strength solution culture (Murashige and Skoog Basal Salt Micronutrient Solution; 10×, liquid, plant cell culture tested; SIGMA Life Science). The plants were watered three times a week. Each pot was enclosed in a growth chamber (Cultibox SG combi 80 × 80 × 160 cm) so the plants could grow in controlled environmental conditions (Figure 2). The chamber air temperature was set at 26°C and remained constant between 24°C and 26°C during the day – night cycle; the extractor fan was equipped with a thermo-regulator (TT125; 125 mm-diameter; max280 MC/H vents), and there was an input-ventilation fan (Blauberg Tubo 100–102 m3/h). The two-fan combination allowed for a steady airflow rate into the growth chamber with a mean air residence time of 60 s. The fan was placed so that air movement did not affect the plants’ movements. Plants were grown with an 11.25-hr photoperiod (5.45 am to 5 pm) under a cool white LED lamp (V-TAC innovative LED lighting, VT-911-100W, Des Moines, IA, USA or 100W Samsung UFO 145 l m/W – LIFUD) that was positioned 57 cm above each seedling (Figure 2). Photosynthetic photon flux density at 57 cm under the lamp in correspondence with the seedlings was 350 μmolph/m2s (quantum sensor LI-190 R, Lincoln, Nebraska, USA). Reflective Mylar® film on the chamber walls allowed for better uniformity in light distribution.

### Procedures

To study the influence of SL mutation during the ascent and attachment behavior of *P. sativum* plants, we capitalized on a paradigm that was successful in characterizing 3D kinematics in pea plants^20,22^; Figure 2). We compared *P. sativum* L. wild-type, *P. sativum rms1–1* (L77 background), *and P. sativum rms3–1* (Torsdag background) plants when reaching toward and grasping a potential support located at a distance of 10 cm (Figure 2).

### Dependent measures

**Figure 3:**
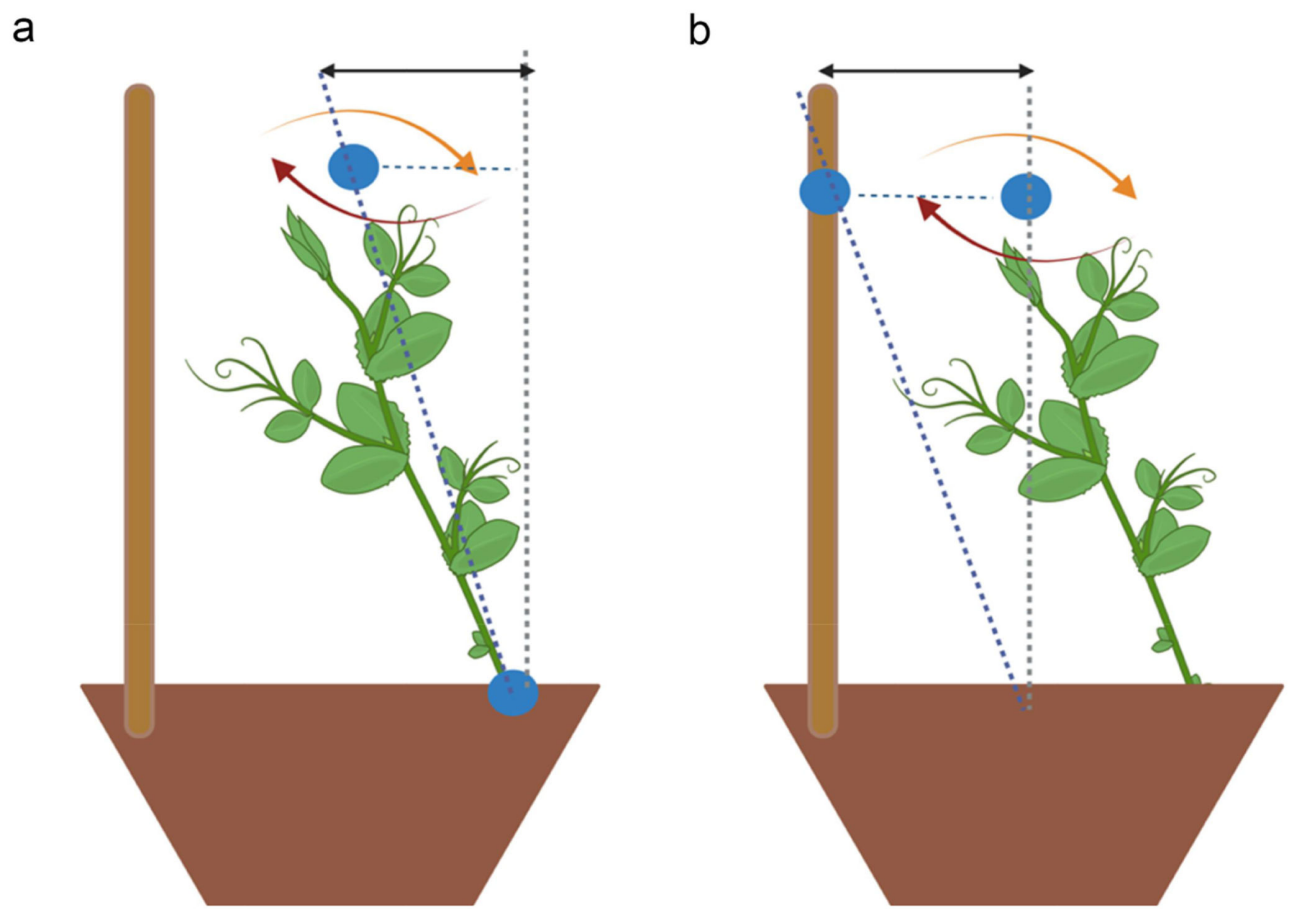
Panel a: graphical representation of the distance between the gravity center of the circumnutation to the origin of the plant as represented by the bidirectional black solid arrow. Panel b: graphical representation of the distance between the gravity center of the circumnutation to the support as represented by the bidirectional black solid arrow.

The dependent variables specifically tailored to test our experimental hypothesis based on previous kinematic studies on approach-to-grasp in pea plants (e.g.,^20,23^ were as follows (i) the spatial trajectories designed by the tip of the tendril; (ii) the tendril’s total duration of circumnutation (min), or the interval between the beginning and the end of the movement of the tendrils (i.e., when the tendrils encountered the support or fell); (iii) the amplitude of the mean velocity of the tendril during circumnutation (mm/min), or the average velocity reached by the tendrils during movement time; (iv) the amplitude of the maximum acceleration of the tendril during circumnutation (mm/min^2^), or the maximum variation of velocity during the movement time; (v) the distance between the gravity center of the circumnutation and the origin of the plant (mm), or the inclination of the stem of the plant to explore the surrounding (Figure 3a); and (vi) the distance between the gravity center of the circumnutation and the support (mm), or the inclination of the plant toward the support (Figure 3b).

### Video recording and registration

For each growth chamber, a pair of RGB-infrared cameras (i.e., IP 2.1 Mpx outdoor varifocal IR 1080P) were placed 110 cm above the ground, spaced at 45 cm to record stereo images of the plant (Figure 2). The cameras were connected via Ethernet cables to a 10-port wireless router (i.e., D-link Dsr-250n) connected via Wi-Fi to a PC, and the frame acquisition and saving processes were controlled by CamRecorder software (Ab.acus s.r.l., Milan, Italy). To maximize the contrast between the peas’ anatomical landmark (e.g., the tendril) and the background, black felt velvet was fixed on some sectors of the boxes’ walls. Each camera’s intrinsic, extrinsic, and lens distortion parameters were estimated using a Matlab Camera Calibrator App. Depth extraction from the single images was conducted by taking 20 pictures of a chessboard (squares’ sides 18 mm, 10 columns, 7 rows) from multiple angles and distances in natural indirect light conditions. For stereo calibration, the same chessboard used for the single-camera calibration process was placed in the middle of the growth chamber. The two cameras took photos to extract the stereo calibration parameters. Via the experimental protocol, the camera synchronously acquired a frame every 3 min (frequency 0.0056 hz). The tendrils developing from the considered node were studied. In cases in which the plant grasped the stimulus, the coiled leaf was analyzed. The initial frame was defined as the frame in which the considered leaf’s tendrils were visible from the apex. The end of the plant’s movement was defined as the frame in which the leaf’s tendrils started to coil around the support or the plant stopped moving or fell. Ad hoc software (SPROUT, Ab.acus s.r. l., Milan, Italy^20^; developed in Matlab and Python was used to identify anatomical points to be investigated via markers and to track their position frame by frame on the images of the two cameras acquired to reconstruct the 3D trajectory of each marker. The markers on the anatomical landmark of interest, namely the tip of the tendril, were inserted post hoc. The tracking procedures were first performed automatically throughout the course of the movement sequence using the Kanade-Lucas-Tomasi algorithm on the frames each camera acquired after distortion removal. The tracking was manually verified by the experimenter, who checked the positions of the markers frame by frame. The 3D trajectory of each tracked marker was computed by triangulating the 2D trajectories obtained from the two cameras.

### Statistical analysis

Statistical analyses were conducted using the frequentist approach. To check the normality of the dataset, we performed a Shapiro Wilk test before starting the formal analysis. Regarding the kinematic measures, we adopted the Kruskal–Wallis nonparametric ANOVA because the dependent variables are not normally distributed. The descriptive statistics, including median, interquartile range (IQR), range, and percentiles (25th, 50th, and 75th) have been calculated. The Kruskal–Wallis test is a nonparametric test that does not require the assumption of normality. The results are displayed by reporting the statistic – that is, the value for the test statistic; df reports the degrees of freedom, and *p* represents the p-value. We also performed the Tukey post hoc test to check the differences among the groups in more detail. The analyses were performed using JASP^39^ nested within the environment R (R Development Core^40^ see used packages: https://jaspstats.org/rpackage-list/). The null hypothesis here is that there is no difference in kinematics between the analyzed conditions. The alternative hypothesis is that there is a difference.

## Results

### Qualitative results

**Figure 4:**
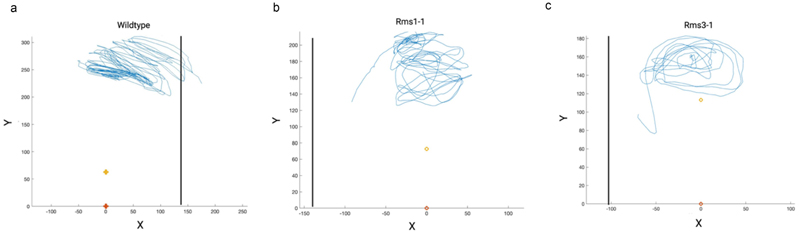
Trajectory of the tendril for a representative plant of each genotype. The black vertical line represents the support. Yellow and orange reference points represent the internode below the tendrils and the origin of the plant (as a reference point), respectively. The blue line represents the trajectories of the tip of the tendril.

Inspecting the trajectories of the tendrils shows a circumnutative kind of movement for all the considered plants. As shown in Figure 4, all the plants performed an expected number of rotations.^20,23^ Interestingly, only the wild-type (Figure 4a; see supplementary material Video S3) oriented its movement toward the support and grasped. The mutant plants fell, given that they were unable to properly detect and grasp the support, as evidenced by following the downward trajectory of the tendril (Figures 4b,c; see supplementary material Video S1, S2).

### Kinematic results

**Figure 5:**
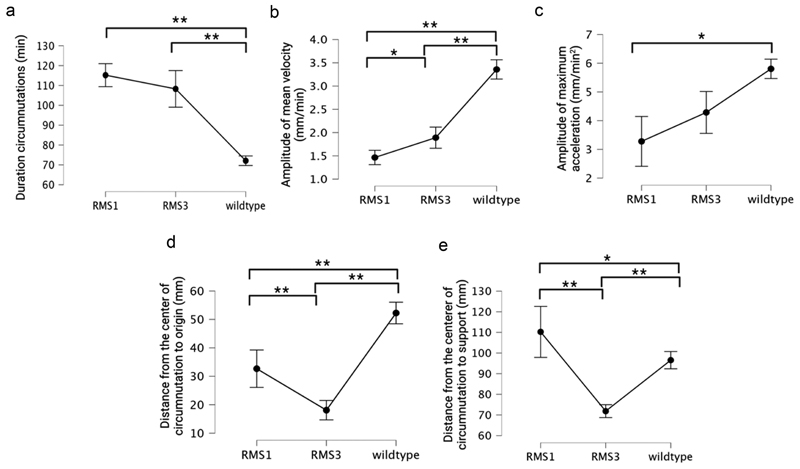
Basic plots with error bars for all the dependent variables measured for the three genotypes, namely *rms1–1, rms3–1*, and wild type. Squared brackets indicate the significative comparison among genotypes. * represents a value for *p* < .005; ** represents a value for *p* < .001.

When looking at the descriptive statistic for the kinematical measures, a substantial difference across genotypes is immediately recognizable (see Table S1 in supplementary material; see Figure 5). For the duration of circumnutations, the wild-type plants circumnutated more quickly than the mutants (*H*[2] = 159.427 min, *p* = <.001; see Figure 5a, Tables S1, S3 in supplementary material). When comparing movement duration between *rms1–1* and *rms3–1*, the latter was faster than the former, although not statistically significantly (see supplementary material Table S3).

Coherently with the pattern observed in movement duration, we observe a faster velocity for the wild-type plants with respect to the mutants (*H*(2) = 134.837 mm/min, *p* = <.001; see Figure 5b,c; see Tables S1, S2 in supplementary material). Both the *rms* mutants moved slower than the wild-type, with the *rms1–1* mutant plants showing the lowest amplitude of mean velocity with respect to the other two genotypes that produces SLs (i.e., *rms3–1* mutant and wild-type; Figure 5b, see also Table S4 in supplementary material). The amplitude of maximum acceleration was lower for the *rms1–1* and *rms3–1* mutants than for the wild type, although this was only statistically significant for *rms1–1* (*H*(2) = 91.726 mm/min^2^, *p* = <.001; see Figure 5c; see also Table S5 in supplementary material) plants. Overall, this kinematical pattern suggests an involvement of SLs in the ability to move and implement faster goal-directed movements. When looking at the spatial characteristics of circumnutation, the distance between the center of the circumnutation to the origin of the plant is greater for the wild-type than the mutants (*H*[2] = 124.609 mm, *p* = <.001; see Figure 5d; see also Tables S1, S2 in supplementary material). Wild-type plants showed a larger inclination from their origin with respect to the *rms1–1* and *rms3–1* (see Figure 5d and Table S6 in supplementary material). Between the two mutants, the difference was lower but still present, with a slightly larger inclination of the *rms1–1* with respect to the *rms3–1* (see Figure 5d and Table S6 in supplementary material). The distance between the center of the circumnutation to the support was different among the three groups of plants (*H*[2] = 68.864, *p* = < .001) with *rms1–1* remaining more distant from the support with respect to *rms3–1* (see Figure 5e and Table S7 in supplementary material). This distance was slightly bigger for the *rms1–1* compared to the wild-type and decisively bigger when considering the *rms3–1* and the wild-type (see Figure 5e and Table S7 in supplementary material).

## Discussion

The aim of the present study is to test whether SLs play a role in determining successful ascent and attachment behavior in pea plants. It provides a comprehensive kinematical account of this behavior in SL mutant plants compared to wild-type plants. In general, the results indicate that mutant plants are unable to locate and grasp a potential support. Their movement appears to be disoriented and much less energized. Building upon the well-established roles of SLs documented in the literature – such as root-to-shoot communication, regulation of stem growth, promotion of secondary branching, and modulation of root and shoot architecture^6–10^—we identify the climbing and attachment behaviors of these plants as a potential lens through which SLs’ influence on plant behavior can be observed and analyzed.

From these assumptions, a clear distinction between the mutants and the wild-type plants is evident when examining the qualitative results of the tendril trajectories. Wild-type plants exhibited a prominent slant toward the support, whereas the *rms* mutant plants displayed an appropriate yet untargeted circumnutative behavior. These trajectories indicate that wild-type plants conclude their behavior with a grasping phase, while *rms* mutants interrupt their circumnutative behavior, failing to clasp the support (Figure 4). This differentiation between *rms* mutants and wild-type plants is even more pronounced in kinematic terms, as outlined below.

When comparing the ascent and attachment behavior of the wild-type plants to that of the mutants (Figure 5), we observe a significantly shorter circumnutation duration for the former with respect to the latter plants. The observation of faster movement for the wild-type plants is supported by the amplitude of mean velocity and acceleration. Both were higher for the wild-type plants than for the *rms1–1* and *rms3–1* mutants. In spatial terms, the distance between the center of circumnutation and the origin of the plant reveals that wild-type plants achieved the greatest distance, reflecting a more exploratory behavior in their surrounding environment compared to the mutant plants. Notably, wild-type plants were the only ones that successfully grasped the support.

Our results show some clear differences between *rms1–1* and *rms3–1* mutants (Figure 5b,d,e), but these results should be treated with some caution since the two mutants are in different genetic backgrounds (L77 and Torsdag) respectively. Thus, the differences between the mutants might reflect fundamental differences in the genetic background, rather than specific differences between *rms1–1* and *rms3–1*. When compared to wild-type, the two mutants are qualitatively similar to each other, and qualitatively different to wild-type. At this time, it is therefore most likely that strigolactone synthesis and signaling play a very similar role in climbing behavior, although further experiments might reveal clearer differences between the two mutants.

These results confirm our first hypothesis that specific mutations could lead to modifications at the behavioral level. For SLs, the effects of mutations have primarily been documented at the morphological level, with modified plants exhibiting differences in shoot branching, leaf blade, and petiole length, leaf senescence, internode elongation, and final height.^41,42^ Here we add to this literature, demonstrating that modifications are also evident at a behavioral level when mutations concerned with SLs occur.

### Understanding the role of strigolactones in climbing behaviour in pea

Our results show that SLs are involved in the climbing behavior of pea plants, but do not identify in what capacity SLs act. There are at least three possible, non-mutually exclusive ways that SLs might be involved in these responses. Firstly, SLs might be involved in the underground perception of the potential support. Plant root exudates have been shown to be involved in the ability of plants to detect obstacles in the soil,^43^ and SLs are exudates that are released and perceived by plants in the soil. The relative dilution of SLs in soil appears to allow plants to perceive both their physical and biological environment).^12,44^ Thus, accumulation of SLs in the proximity of the underground part of a potential support could be a mechanism by which plants detect that support. Therefore mutants in SL synthesis or perception would not be able to detect the presence of a potential support.

Alternatively, the role of SLs might be more with regard to the root-to-shoot communication of the presence of a potential support. SLs are well established to act as root-to-shoot signals by elegant grafting experiments, although the precise details of their movement are still unclear (reviewed in ^30^). The presence of a potential support detected underground might lead to increase root-to-shoot SL transport, triggering an increase in the speed and inclination of circumnutation observed in wild-type plants. Conversely, mutants lacking in SL synthesis or signaling would not be able to increase root-to-shoot transport or respond to such an increase, respectively.

A third possibility is that the role of SLs is permissive rather than instructive. The amplitude of mean velocity serves as an index of strength, fluency, and readiness in motor behavior. Key physiological characteristics necessary for the fluency of movement in climbing plants include stem flexibility and organ stability (i.e., secondary growth,^45^ along with the production of biomass to facilitate these phenotypic modifications.^45^ Efficient utilization of biomass necessitates finding an optimal balance between primary and secondary growth; whereas longer stems enhance exploration, increased thickness improves resistance to mechanical stress.^45^ SLs could play a crucial role in this, given their known involvement in the secondary growth of shoots.^33^ This process is critical for stem elongation, facilitating flexed movement and a wider rotation of the tendrils toward the surrounding environment. Agusti et al.^33^ demonstrated that SL-deficient mutants exhibit reduced secondary growth, which might explain why SL mutants, despite their inclination to explore their surroundings, were unable to successfully reach the support. Their shorter stems and reduced secondary growth might therefore have hindered their ability to effectively orient themselves toward the wooden pole.

## Conclusion

The present study investigated SLs’ role in ascent and attachment behaviors in pea plants. It provides a quantitative analysis of distinct kinematic movement patterns in plants capable and incapable of SL production or perception. Importantly, this research opens new avenues for exploring SLs’ role in plant behavior. This marks a critical step forward in recognizing plants as behavioral organisms, with the potential to advance the study of behavior, genetics, and epigenetics in plant systems.

It should be noted, however, that the present study is not without limitations. To start with, a limitation of the present study is its focus on a single plant species, along with the lack of online measurement of SL emission by wild-type plants during movement execution. This could be a crucial point for further studies in this area of research by quantifying SL emissions from wild-type plants during ascent and attachment behavior using mass spectrometry techniques. This might allow deeper investigations under diverse ecological conditions to gain further insights on the functional equilibrium and interactivity between plants’ modules driven by SLs above and below ground. Future experiments should ascertain whether the avoidance of the putative SLs accumulation at the underground site of the support is sufficient to modify the tendril movements. This could be done by studying SLs mutant plants when only the underground part of the support is available. Finally, an analysis of tendrils’ cell elongation in wild type and SLs mutants could prove valuable in evaluating the potential influence of SLs on cell growth, which may in turn affect movement.

## Supplementary Material

Supporting Information

## Data Availability

The data that support the findings of this study are openly available in ZENODO at http://doi.org/10.5281/zenodo.14274244.
