# Supplementary material for: Do strigolactones play a role in the ascent and attachment behavior of Pisum sativum?
Source: Plant Signal Behav. 2024 Dec 30;20(1):2447455. doi: 10.1080/15592324.2024.2447455 (PMC7617743; doi:10.1080/15592324.2024.2447455)
Supplement: Supplementary material Tables.docx [file KPSB_A_2447455_SM3761.docx]

| Table S1. Descriptive Statistics for the rms1-1, rms3-1 mutant plants and the wildtype plants. | | | | | | | | | | | | | | | | |  |
| --- | --- | --- | --- | --- | --- | --- | --- | --- | --- | --- | --- | --- | --- | --- | --- | --- | --- |
|  | |  | **Median** | | **IQR** | | | **Range** | **25th percentile** | | | **50th percentile** | | **75th percentile** | | |  |
| Duration of circumnutations |  | RMS-1 |  | 108.000 |  | 36.000 |  | 168.000 |  | 96.000 |  | 108.000 |  | | 132.000 |  | |
|  |  | RMS3-1 |  | 96.000 |  | 54.750 |  | 255.000 |  | 78.000 |  | 96.000 |  | | 132.750 |  | |
|  |  | wildtype |  | 69.000 |  | 21.000 |  | 90.000 |  | 60.000 |  | 69.000 |  | | 81.000 |  | |
| Amplitude of mean velocity |  | RMS1-1 |  | 1.324 |  | 1.459 |  | 5.045 |  | 0.708 |  | 1.324 |  | | 2.167 |  | |
|  |  | RMS3-1 |  | 2.042 |  | 2.059 |  | 4.505 |  | 0.773 |  | 2.042 |  | | 2.832 |  | |
|  |  | wildtype |  | 3.202 |  | 2.506 |  | 6.247 |  | 2.091 |  | 3.202 |  | | 4.598 |  | |
| Amplitude of maximum acceleration |  | RMS1-1 |  | 0.264 |  | 0.234 |  | 13.468 |  | 0.143 |  | 0.264 |  | | 0.377 |  | |
|  |  | RMS3-1 |  | 0.384 |  | 0.301 |  | 6.063 |  | 0.217 |  | 0.384 |  | | 0.518 |  | |
|  |  | wildtype |  | 0.660 |  | 0.650 |  | 2.204 |  | 0.355 |  | 0.660 |  | | 1.006 |  | |
| Distance from the center of circumnutations to the origin of the plant |  | RMS1-1 |  | 19.069 |  | 25.842 |  | 147.326 |  | 9.166 |  | 19.069 |  | | 35.008 |  | |
|  |  | RMS3-1 |  | 10.856 |  | 21.708 |  | 66.869 |  | 5.217 |  | 10.856 |  | | 26.925 |  | |
|  |  | wildtype |  | 45.531 |  | 48.178 |  | 105.693 |  | 28.723 |  | 45.531 |  | | 76.900 |  | |
| Distance from the center of circumnutations to the support |  | RMS1-1 |  | 83.989 |  | 23.530 |  | 307.472 |  | 76.297 |  | 83.989 |  | | 99.827 |  | |
|  |  | RMS3-1 |  | 69.539 |  | 19.138 |  | 76.994 |  | 60.596 |  | 69.539 |  | | 79.734 |  | |
|  |  | wildtype |  | 92.811 |  | 28.093 |  | 153.565 |  | 79.410 |  | 92.811 |  | | 107.502 |  | |

| \| Table S2. Kruskal-Wallis Test for the dependent measures of interest comparing rms1-1, rms3-1 and wildtype groups. \| \| \| \| \| \| --- \| --- \| --- \| --- \| --- \| \| **Dependent variables** \| \| **Statistic** \| **df** \| **p** \| \| Duration of circumnutations \|  \| 159.427 2 < .001 \| \| \| \| Amplitude of mean velocity \|  \| 134.837 2 < .001 \| \| \| \| Amplitude of maximum acceleration \|  \| 91.726 2 < .001 \| \| \| \| Distance from the center of circumnutations to the origin of the plant \|  \| 124.609 2 < .001 \| \| \| \| Distance from the center of circumnutations to the support \|  \| 68.864 2 < .001 \| \| \|  \| Table S3. Post Hoc Comparisons – Duration of circumnutations \| \| \| \| \| \| \| \| \| \| \| \| \| --- \| --- \| --- \| --- \| --- \| --- \| --- \| --- \| --- \| --- \| --- \| --- \| \|  \| \|  \| \| **Mean Difference** \| \| **SE** \| \| **t** \| \| **p_tukey_** \| \| \| RMS1 \|  \| RMS3 \|  \| 6.920 \|  \| 4.153 \|  \| 1.667 \|  \| 0.219 \|  \| \| RMS1 \|  \| wildtype \|  \| 43.102 \|  \| 3.517 \|  \| 12.257 \|  \| < .001 \|  \| \| RMS3 \|  \| wildtype \|  \| 36.181 \|  \| 3.788 \|  \| 9.551 \|  \| < .001 \|  \| \|  \| \| \| \| \| \| \| \| \| \| \| \| \| *Note.*  P-value adjusted for comparing a family of 3 \| \| \| \| \| \| \| \| \| \| \| \|   Table S4. Post Hoc Comparisons – Amplitude of mean velocity | | | | | | | | | | | |
| --- | --- | --- | --- | --- | --- | --- | --- | --- | --- | --- | --- | --- | --- | --- | --- | --- | --- | --- | --- | --- | --- | --- | --- | --- | --- | --- | --- | --- | --- | --- | --- | --- | --- | --- | --- | --- | --- | --- | --- | --- | --- | --- | --- | --- | --- | --- | --- | --- | --- | --- | --- | --- | --- | --- | --- | --- | --- | --- | --- | --- | --- | --- | --- | --- | --- | --- | --- | --- | --- | --- | --- | --- | --- | --- | --- | --- | --- | --- | --- | --- | --- | --- | --- | --- | --- | --- | --- | --- | --- | --- | --- | --- | --- | --- | --- | --- | --- | --- | --- | --- | --- | --- | --- | --- | --- | --- | --- | --- | --- | --- | --- | --- | --- | --- | --- | --- | --- | --- | --- | --- | --- | --- | --- | --- | --- | --- | --- | --- | --- | --- |
|  | |  | | **Mean Difference** | | **SE** | | **t** | | **p_tukey_** | |
| RMS1 |  | RMS3 |  | -0.426 |  | 0.169 |  | -2.520 |  | 0.032 |  |
| RMS1 |  | wildtype |  | -1.892 |  | 0.143 |  | -13.230 |  | < .001 |  |
| RMS3 |  | wildtype |  | -1.467 |  | 0.154 |  | -9.519 |  | < .001 |  |
|  | | | | | | | | | | | |
| *Note.*  P-value adjusted for comparing a family of 3 | | | | | | | | | | | |

| Table S5. Post Hoc Comparisons – Amplitude of maximum acceleration | | | | | | | | | | | |
| --- | --- | --- | --- | --- | --- | --- | --- | --- | --- | --- | --- |
|  | |  | | **Mean Difference** | | **SE** | | **t** | | **p_tukey_** | |
| RMS1 |  | RMS3 |  | -0.142 |  | 0.112 |  | -1.270 |  | 0.413 |  |
| RMS1 |  | wildtype |  | -0.299 |  | 0.095 |  | -3.149 |  | 0.005 |  |
| RMS3 |  | wildtype |  | -0.156 |  | 0.102 |  | -1.531 |  | 0.277 |  |
|  | | | | | | | | | | | |
| *Note.*  P-value adjusted for comparing a family of 3 | | | | | | | | | | | |

| Table S6. Post Hoc Comparisons – Distance from the center of circumnutations to the origin of the plant | | | | | | | | | | | |
| --- | --- | --- | --- | --- | --- | --- | --- | --- | --- | --- | --- |
|  | |  | | **Mean Difference** | | **SE** | | **t** | | **p_tukey_** | |
| RMS1 |  | RMS3 |  | 14.579 |  | 3.871 |  | 3.766 |  | < .001 |  |
| RMS1 |  | wildtype |  | -19.582 |  | 3.278 |  | -5.974 |  | < .001 |  |
| RMS3 |  | wildtype |  | -34.161 |  | 3.531 |  | -9.675 |  | < .001 |  |
|  | | | | | | | | | | | |
| *Note.*  P-value adjusted for comparing a family of 3 | | | | | | | | | | | |

| Table S7. Post Hoc Comparisons – Distance from the center of circumnutations to the support | | | | | | | | | | | |
| --- | --- | --- | --- | --- | --- | --- | --- | --- | --- | --- | --- |
|  | |  | | **Mean Difference** | | **SE** | | **t** | | **p_tukey_** | |
| RMS1 |  | RMS3 |  | 38.386 |  | 5.885 |  | 6.523 |  | < .001 |  |
| RMS1 |  | wildtype |  | 13.700 |  | 4.983 |  | 2.749 |  | 0.017 |  |
| RMS3 |  | wildtype |  | -24.686 |  | 5.368 |  | -4.598 |  | < .001 |  |
|  | | | | | | | | | | | |
| *Note.*  P-value adjusted for comparing a family of 3 | | | | | | | | | | | |
